# Genome replication engineering assisted continuous evolution (GREACE) to improve microbial tolerance for biofuels production

**DOI:** 10.1186/1754-6834-6-137

**Published:** 2013-09-27

**Authors:** Guodong Luan, Zhen Cai, Yin Li, Yanhe Ma

**Affiliations:** 1 CAS Key Laboratory of Microbial Physiological and Metabolic Engineering, Institute of Microbiology, Chinese Academy of Sciences, No. 1 West Beichen Road, Chaoyang District, Beijing 100101, China; 2 University of Chinese Academy of Sciences, Beijing 100049, China; 3 State Key Laboratory of Microbial Resources, Institute of Microbiology, Chinese Academy of Sciences, Beijing 100101, China

**Keywords:** Mutagenesis coupled-with Selection, GREACE, Genome replication engineering, Continuous evolution, Strain improvement

## Abstract

**Background:**

Microbial production of biofuels requires robust cell growth and metabolism under tough conditions. Conventionally, such tolerance phenotypes were engineered through evolutionary engineering using the principle of “Mutagenesis followed-by Selection”. The iterative rounds of mutagenesis-selection and frequent manual interventions resulted in discontinuous and inefficient strain improvement processes. This work aimed to develop a more continuous and efficient evolutionary engineering method termed as “Genome Replication Engineering Assisted Continuous Evolution” (GREACE) using “Mutagenesis coupled-with Selection” as its core principle.

**Results:**

The core design of GREACE is to introduce an *in vivo* continuous mutagenesis mechanism into microbial cells by introducing a group of genetically modified proofreading elements of the DNA polymerase complex to accelerate the evolution process under stressful conditions. The genotype stability and phenotype heritability can be stably maintained once the genetically modified proofreading element is removed, thus scarless mutants with desired phenotypes can be obtained.

Kanamycin resistance of *E. coli* was rapidly improved to confirm the concept and feasibility of GREACE. Intrinsic mechanism analysis revealed that during the continuous evolution process, the accumulation of genetically modified proofreading elements with mutator activities endowed the host cells with enhanced adaptation advantages. We further showed that GREACE can also be applied to engineer n-butanol and acetate tolerances. In less than a month, an *E. coli* strain capable of growing under an n-butanol concentration of 1.25% was isolated. As for acetate tolerance, cell growth of the evolved *E. coli* strain increased by 8-fold under 0.1% of acetate. In addition, we discovered that adaptation to specific stresses prefers accumulation of genetically modified elements with specific mutator strengths.

**Conclusions:**

We developed a novel GREACE method using “Mutagenesis coupled-with Selection” as core principle. Successful isolation of *E. coli* strains with improved n-butanol and acetate tolerances demonstrated the potential of GREACE as a promising method for strain improvement in biofuels production.

## Background

**Figure 1:**
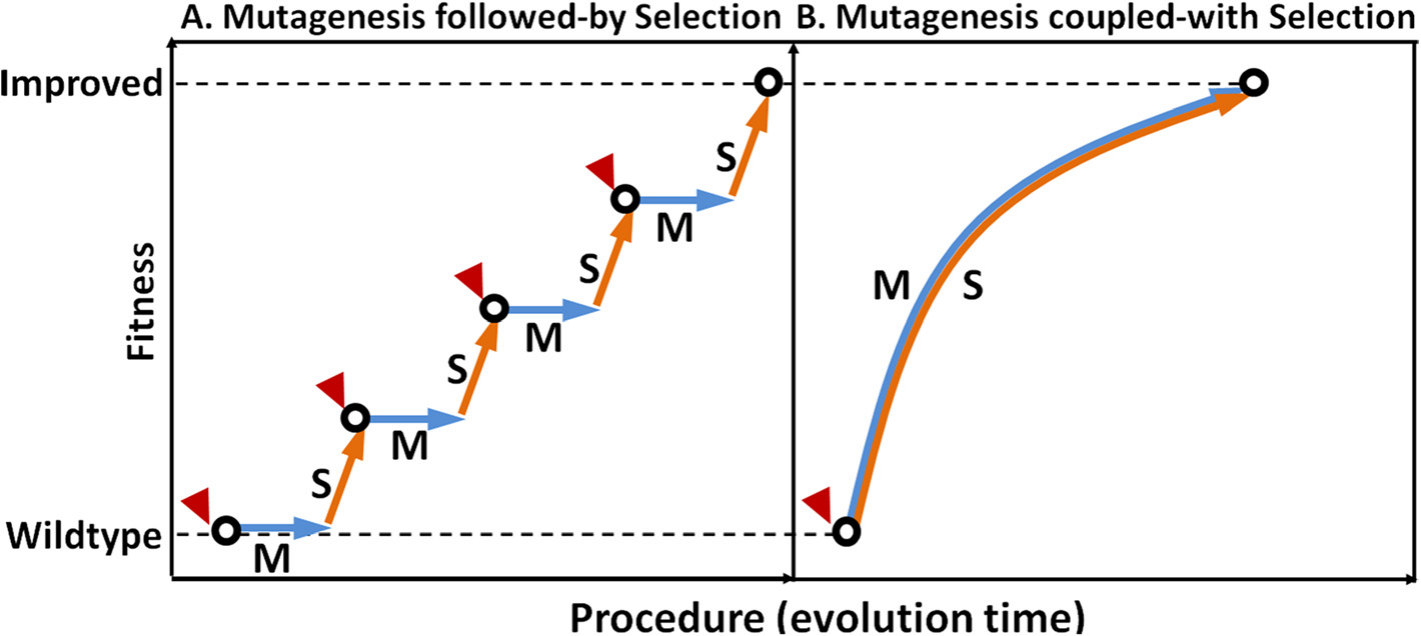
**Comparisons between the principle of “Mutagenesis followed-by Selection” and the principle of “Mutagenesis coupled-with Selection”. (A)** Traditional “Mutagenesis followed-by Selection” principle was usually performed by iterative rounds of mutagenesis and selection. Exogenous mutagens or genetic manipulations were required for mutagenesis and the following selection manipulations isolated cells with improved phenotypes, which could be used in next rounds of “mutagenesis-selection”. **(B)** As for “Mutagenesis coupled-with Selection” principle, the two steps are synchronized, so that iterative and lengthy manual interventions are greatly simplified, leading to a continuous and efficient strain improvement process. “S” represents for “selection”, “M” represents for “mutagenesis”, and red triangles represent for manual interventions such as mutagen treatments or genetic manipulations or selections for improvement phenotypes.

Efficient microbial production of biofuels from renewable resources requires robust cell growth and stable metabolism under tough industrial conditions, represented by inhibitory components in substrates and toxic products [1,2]. Microbial tolerance to these inhibitory environmental factors is a complex phenotype usually controlled by multiple genes [3,4], and thus is difficult to be engineered by targeted metabolic engineering approaches [5]. Instead, such complex phenotypes can be more effectively improved by evolutionary engineering approaches [6]. Examples of evolutionary engineering include successive passage for metabolic evolution [7-9], physical and chemical mutagenesis [10], global transcription machinery engineering [2,11], artificial transcription factors engineering [12,13], and ribosome engineering [14]. All these methods use “Mutagenesis followed-by Selection” as core principle, meaning that firstly introducing genetic diversity by spontaneous mutations, exogenous mutagens, or genetic perturbations, followed by selection of desired phenotypes [6]. Using such methods, iterative rounds of mutagenesis-selection and frequent manual interventions are often required, resulting in discontinuous and inefficient strain improvements, as shown in Figure 1A.

To address the discontinuity of the existing evolutionary engineering approaches and improve the engineering efficiency, we devised a novel method termed as “Genome Replication Engineering Assisted Continuous Evolution, GREACE”, which uses “Mutagenesis coupled-with Selection” (Figure 1B) as its core principle. The key element of this novel method is to introduce *in vivo* continuous mutagenesis mechanisms into microbial cells that are subsequently subjected to continuous selective conditions. Mutagenesis and selection can therefore be coupled to minimize manual interventions, thus providing possibilities to develop a continuous and efficient phenotypes-improving process (Figure 1B). Practically, *in vivo* continuous mutagenesis can be achieved by introducing genetic perturbations into genome replication machinery so as to trigger inaccurate genome replications. Hypermutable cells with significant genome diversities can therefore be obtained. Offspring cells with mutated genomes that survived the increased selective pressures will be selected during the continuous enrichment processes, and the genomic feature of evolved cells with improved phenotypes can be stably maintained once the elements triggering the inaccurate genome replication are removed from the individually isolated cells.

In this work, we proved the concept of GREACE and tested the efficiency of GREACE using *Escherichia coli* as a model. Genetically modified proofreading elements of the DNA polymerase complex (ϵ subunit encoded by *dnaQ* gene) were used to trigger perturbations on genome replication for *in vivo* continuous mutagenesis. We firstly proved the feasibility and intrinsic mechanisms of GREACE by improving kanamycin resistance of *E. coli*. Secondly we show that n-butanol and acetate tolerances of *E. coli*, two important microbial tolerance characteristics, can also be efficiently improved using this method. *E. coli* mutants obtained through the GREACE process showed significantly improved tolerances to n-butanol and acetate, demonstrating the potential of GREACE as an effective and universal approach to improve tough physiological traits required by biofuels production. Furthermore, we discovered a phenomenon that adapting to specific environments calls for specific genetically modified proofreading elements, which may provide insights into understanding and application of evolutionary engineering for strain improvement.

## Results

### Procedure of the “Genome Replication Engineering Assisted Continuous Evolution”

**Figure 2:**
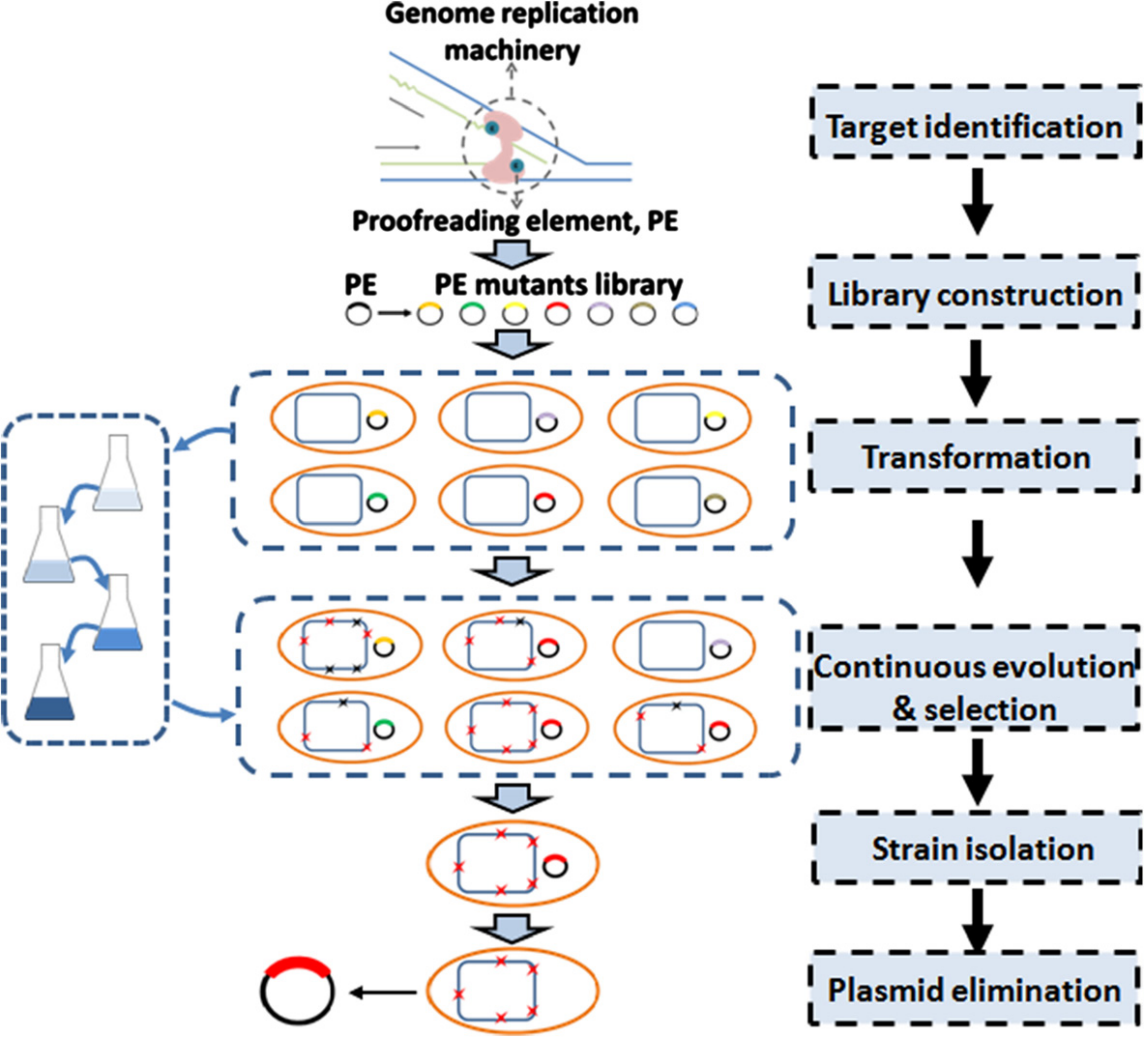
**Flowchart of the Genome Replication Engineering Assisted Continuous Evolution (GREACE) method.** Proofreading element (PE) of the genome replication machinery was selected to generate a mutant library (PEM-lib). For phenotype-improvement, PEM-lib was transformed into host cells, and cultivated in conditions with gradually increased selective strengths (media in flasks with colours from light blue to dark blue). Evolved strains with the most beneficial and adaptive mutation accumulations (red sparks represented for beneficial mutations and black sparks for detrimental mutations) would show the best adaption advantages and dominate the PEM-lib populations. Genetically modified PE mutant would be eliminated from the evolved strains to stabilize the obtained genotypes and phenotypes.

The core idea of GREACE is to introduce genetic perturbations into genome replication machinery, so that *in vivo* continuous mutagenesis can be coupled with simultaneous phenotypes selection. A typical flowchart of GREACE is described in Figure 2. To achieve an inaccurate genome replication, genetic perturbations on genome replication machinery are generated by constructing a mutant library of a proofreading element (PE) gene, designated as PEM-lib. Subsequently, the PEM-lib is transformed into the wildtype strain. Inaccurate genome replication will be triggered in cells containing PEs with decreased proofreading activities, thus continuously generating offspring cells containing different genomic mutations. Notably, the diversity of the PEM-lib will help to generate cells with various genome replication mutation rates and mutation type preferences, ensuring the highest genomic diversity in offspring cells. When cells containing a genetically modified PEs are cultivated under gradually increased selective pressures, only the offsprings with accumulated adaptive mutations can survive and thus to be finally selected. Most importantly, the genome replication will return to regular state once the genetically modified PE is eliminated from the selected cells, so that the genomic features and the evolved phenotypes can be stably maintained.

### Proof of concept for GREACE by engineering kanamycin resistance of *E. coli*

To prove the concept of GREACE, we took *E. coli* as a model strain and selected kanamycin resistance as a phenotype for testing. Among the various cellular proofreading elements including the genes responsible for base selection, exonucleolytic editing, MMR (Methyl-directed Mismatch Repair), and DNA repair [15-18], we chose the *dnaQ* gene which encodes the ϵ subunit of *E. coli* DNA polymerase III. This subunit is the only one with 3′->5′ exonuclease activity in DNA polymerase III, the major DNA polymerase of *E. coli* genome replication, and it was reported to guarantee both the polymerization and error-editing process of DNA replication. Moreover, the strongest mutator phenotypes that have been known were resulted from deficiencies of the *dnaQ* gene [17,19,20]. Thus, *dnaQ* was selected as the proofreading element to be engineered.

**Figure 3:**
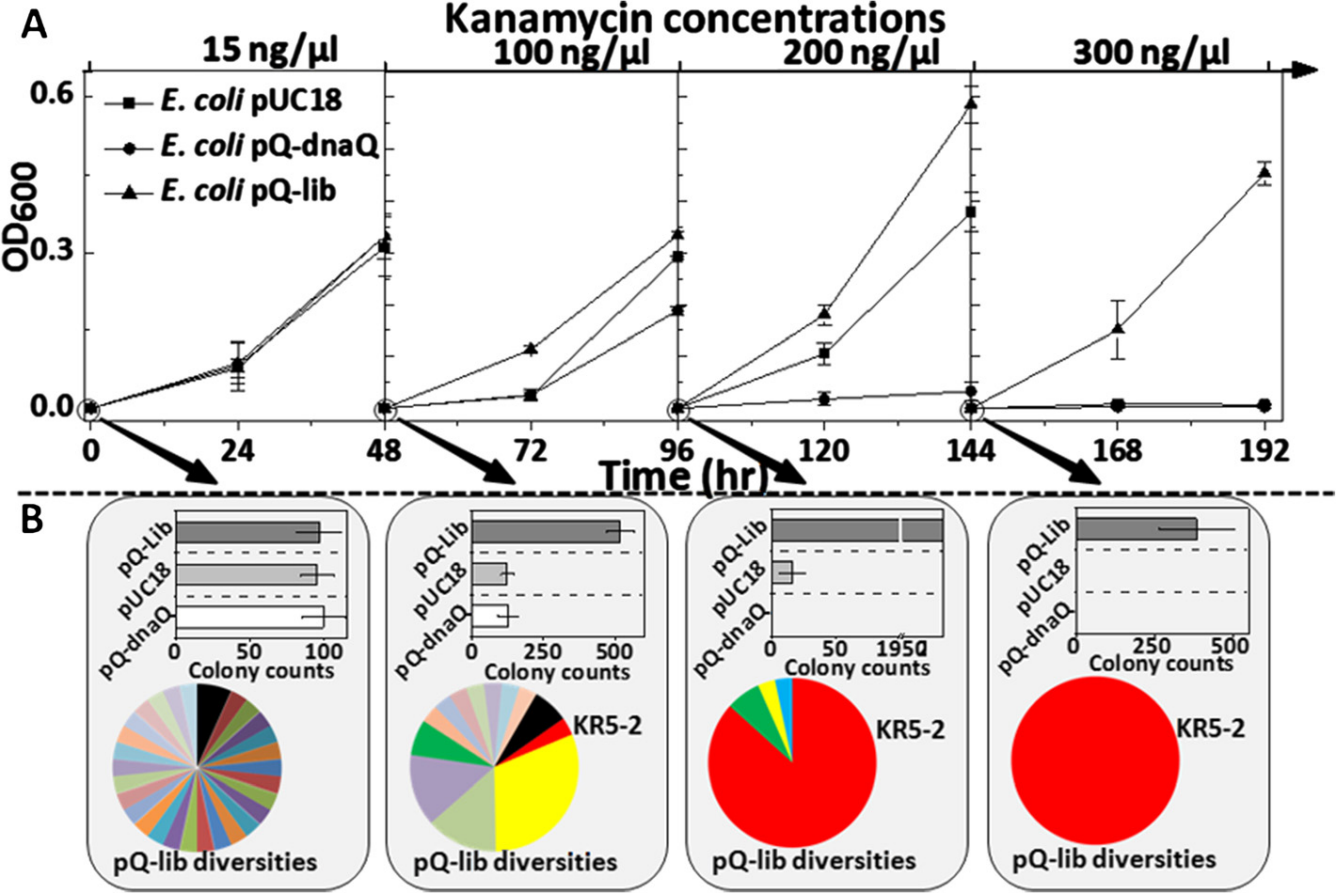
**Engineering kanamycin resistance of *E. coli *with GREACE. (A)***E. coli* cells transformed with pQ-lib, pUC18, and pQ-dnaQ were pre-cultivated in LB medium and then 1:100 serial diluted in gradually increased kanamycin concentrations, from Kan15 to Kan300. Error bars presented the standard deviation of growth analysis for 3 independent evolution experiments. **(B)** Cells carrying the above three plasmids were sampled at the end of the kanamycin resistance evolution process. Appropriate 10^8^ cells were spread on plates with kanamycin concentration of the next level, and cultivated for colony counting. Pie charts represent the proportions of *dnaQ* mutants in 30 randomly selected colonies of *E. coli* cells carrying the pQ-lib at the end of cultivations.

A mutant library of *dnaQ* gene (pQ-lib) with the size of 10^6^ and average mutation rates of 2–3 amino acids per gene product, was constructed by error-prone PCR. pQ-lib, together with the control vectors pQ-dnaQ (carrying wildtype *dnaQ*), and the empty vector pUC18, was transformed into *E. coli* cells, respectively. All cultures were serially transferred in media containing serial concentrations of kanamycin (15 μg/ml, 100 μg/ml, 200 μg/ml, and 300 μg/ml; X μg/ml kanamycin will be designated as KanX). As shown in Figure 3A, no obvious growth differences were observed among three groups grown in Kan15. Upon increasing the kanamycin concentrations, growth and adaptation advantage of the pQ-lib group became obvious. Finally, the pQ-lib group was able to grow in Kan300, while the pUC18 and pQ-dnaQ groups failed to grow in Kan200 and Kan100, respectively, suggesting the pQ-lib group had a stronger adaptability.

To understand why pQ-lib group showed such adaptation advantages, cultures containing 10^8^ cells were collected from pQ-lib, pUC18, and pQ-dnaQ carrying strains grown at each kanamycin concentration and spread onto plates containing higher kanamycin concentrations (cells from Kan0, Kan15, Kan100, and Kan200 liquid culture were spread on plates containing Kan15, Kan100, Kan200, and Kan300, respectively). As shown in Figure 3B, growths of the three groups in Kan0 did not make difference on generation of Kan15 resistant colonies. Growth of the pQ-lib group in Kan15 liquid culture generated more Kan100 resistant colonies than that of the controls, similar results were observed for pQ-lib group grown in Kan100 and Kan200. This suggested that pQ-lib group was able to generate kanamycin-resistant cells more rapidly.

To investigate the dynamic changes of the diversity of pQ-lib during the evolution process, 30 colonies of the pQ-lib group were randomly picked at the end of cultivations at each kanamycin concentration. Plasmids were extracted from these colonies and the *dnaQ* mutants therein were sequenced. As shown in the pie charts of Figure 3B, the diversity of pQ-lib decreased dramatically, and one mutant termed as *dnaQ* KR5-2 was enriched, from undetectable level in Kan0 to 100% in Kan200.

To verify whether the observed adaptation advantages were endowed by *dnaQ* KR5-2, plasmid pQ-dnaQ-KR5-2 was retransformed into fresh *E. coli* cells and cultivated under kanamycin stress. As expected, *E. coli* cells carrying *dnaQ* KR5-2 indeed exhibited increased evolution speed and adaptability. In Kan15 and Kan100, the *dnaQ* KR5-2 carrying strain grew to OD_600_ of 1.02 (±0.07) and 0.28 (±0.05) after cultivation for 48 hours, which were 3–4 fold higher than those of the two control strains carrying pQ-dnaQ and pUC18 (Additional file 1: Figure S1). These results confirmed that adaptation advantages of *dnaQ* KR5-2 under stressful conditions.

To quantitatively evaluate and compare the rates of generating genomic DNA mutations (termed as mutation rates for short) by different *dnaQ* mutants, we chose the widely used rifamycin resistant colony generation frequency as an indirect indicator [21-23]. Mutation rates determination based on this indicator showed that *E. coli* cells carrying pQ-dnaQ-KR5-2 exhibited a 317-fold increased mutation rate than that of the wildtype control, confirming that introducing genetic perturbation into genome replication machinery indeed triggered inaccurate genome replication.

According to the design principle of GREACE, phenotypes obtained by GREACE should be stable and heritable. To confirm the stability of kanamycin resistance, plasmid pQ-dnaQ-KR5-2 was eliminated from the kanamycin resistant strain *E. coli* KR1. We found that the kanamycin resistance could be well maintained after plasmid elimination (Additional file 1: Figure S2).

### GREACE can be successfully applied to engineer n-butanol and acetate tolerances

Recently, *E. coli* has been proved to be a promising host for biobutanol production [24-26]. However, n-butanol tolerance of *E. coli* is a bottleneck hampering further increase of n-butanol titer, as n-butanol is highly toxic to microbial cells. Many efforts have been made to improve and analyze butanol tolerance of *E. coli*[13,27-30]. We then wanted to test if GREACE can be used to improve n-butanol tolerance of *E. coli*, a tougher physiological trait comparing with kanamycin resistance. We applied a similar procedure to engineering n-butanol tolerance – *E. coli* cells carrying pQ-lib were serially transferred in media containing gradually increased n-butanol concentrations. Considering that improvement of complex traits such as n-butanol tolerance might require accumulation of much more adaptive mutations [31,32], the pQ-lib carrying cells were transferred in each n-butanol concentrations for three times, upon full growth, before transferring to the next higher butanol concentration.

**Figure 4:**
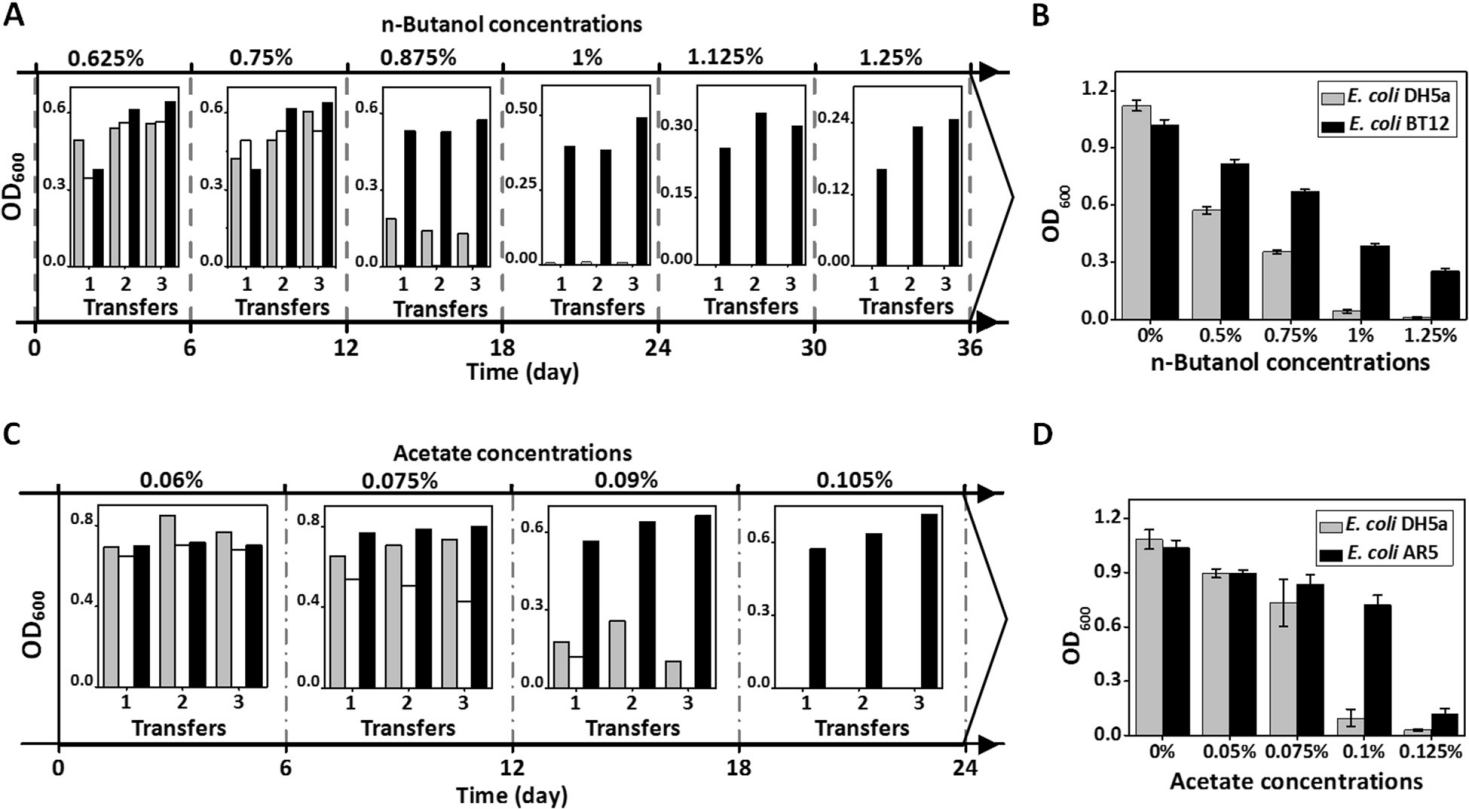
**Application of GREACE to improve n-butanol tolerance and acetate tolerance of *E. coli*.***E. coli* cells carrying pQ-lib (white bars), pUC18 (light-grey bars), and pQ-dnaQ (black bars) were cultivated in gradually increased n-butanol or acetate concentrations for acquiring improved tolerance. On each n-butanol or acetate concentration, 3 transfers were repeated before inoculated to the next concentration. n-Butanol or acetate tolerance of the finally isolated *E. coli* mutant strains (BT12 and AR5) were compared with the wildtype control. **(A)** Evolution process of *E. coli* for n-butanol tolerance. **(B)** Growth assay of *E. coli* BT12 and *E. coli* DH5α under serial n-butanol concentrations. **(C)** Evolution process of E. coli for acetate tolerance. **(D)** Growth assay of *E. coli* AR5 and *E. coli* DH5α under serial acetate concentrations. On each n-butanol or acetate concentration, 3 transfers were repeated before inoculated to the next concentration. As for **(A)** and **(C)** the bars represented for the final optical densities (OD_600_) of each transfer and cultivation.

Figure 4A showed that pQ-lib carrying cells exhibited a better adaptability to the increased n-butanol concentrations. After continuous cultivations and selections, pQ-lib group could grow in medium containing n-butanol concentration of 1.25% (vol/vol, the same below), while n-butanol concentrations of 0.875% and 0.75% were lethal to the *E. coli* cells carrying pUC18 and pQ-dnaQ, respectively. The *dnaQ* mutant dominating the n-butanol tolerance evolution process was isolated and termed as *dnaQ* BR1. Further analysis revealed that *dnaQ* BR1 endowed the host with a 2839-fold increased mutation rate, much higher than that of *dnaQ* KR5-2 selected under kanamycin stress.

After plasmid elimination, an *E. coli* strain, designated as BT12, was obtained. Growth assay revealed that *E. coli* BT12 performed growth advantages upon n-butanol challenge (Figure 4B). At an n-butanol concentration of 0.75%, growth of *E. coli* BT12 reached a doubled OD_600_ value after 48 hours cultivation compared with the wild type strain. When n-butanol concentration reached 1.25%, no growth was detected for the wildtype, while *E. coli* BT12 was still able to grow (25% OD_600_ value of that under no n-butanol stress). n-Butanol shock experiments revealed that *E. coli* BT12 showed a higher cellular stability and tolerance under an extreme lethal n-butanol concentration. After exposure to 2% n-butanol for 1 hour, the survival rate of *E. coli* BT12 was over 100-fold higher than that of the wildtype cells (Additional file 1: Figure S3). We further tested the stability of the n-butanol tolerance of *E. coli* BT12. After frozen at −80°C for 2 weeks and serially transferred for 30 passages, the n-butanol tolerance of *E. coli* BT12 could be well maintained, indicating that good genetic stability and traits heritability can be retained upon GREACE (Additional file 1: Figure S4).

Acetate tolerance is another important trait for biofuels production. Concentrated acetate in hemicelluloses hydrolysates severely inhibited growth and metabolism of microbial cells, thus restricting conversion and production efficiency [33-35]. GREACE was also successfully applied to improve *E. coli* tolerance to acetate. As shown in Figure 4C, the inhibitory effects of acetate on the evolved cells were sharply reduced after transferring *E. coli* cells carrying pQ-lib in gradually increased acetate concentrations for 24 days. pQ-lib carrying cells successfully adapted to an acetate concentration of 0.105% (vol/vol, the same below), while the control could hardly grow in a lower concentration of 0.09%. After plasmid elimination, an *E. coli* mutant AR5 was obtained. AR5 cells showed an 8-fold increased OD_600_ as compared to that of the wildtype strain when grown in the presence of 0.1% acetate (Figure 4D). The *dnaQ* mutant dominating the acetate tolerance evolution process was isolated and termed as AR6-1, endowing host *E. coli* cells with an 87-fold increased mutation rate.

### Characterization of *dnaQ* mutants selected from different conditions

**Figure 5:**
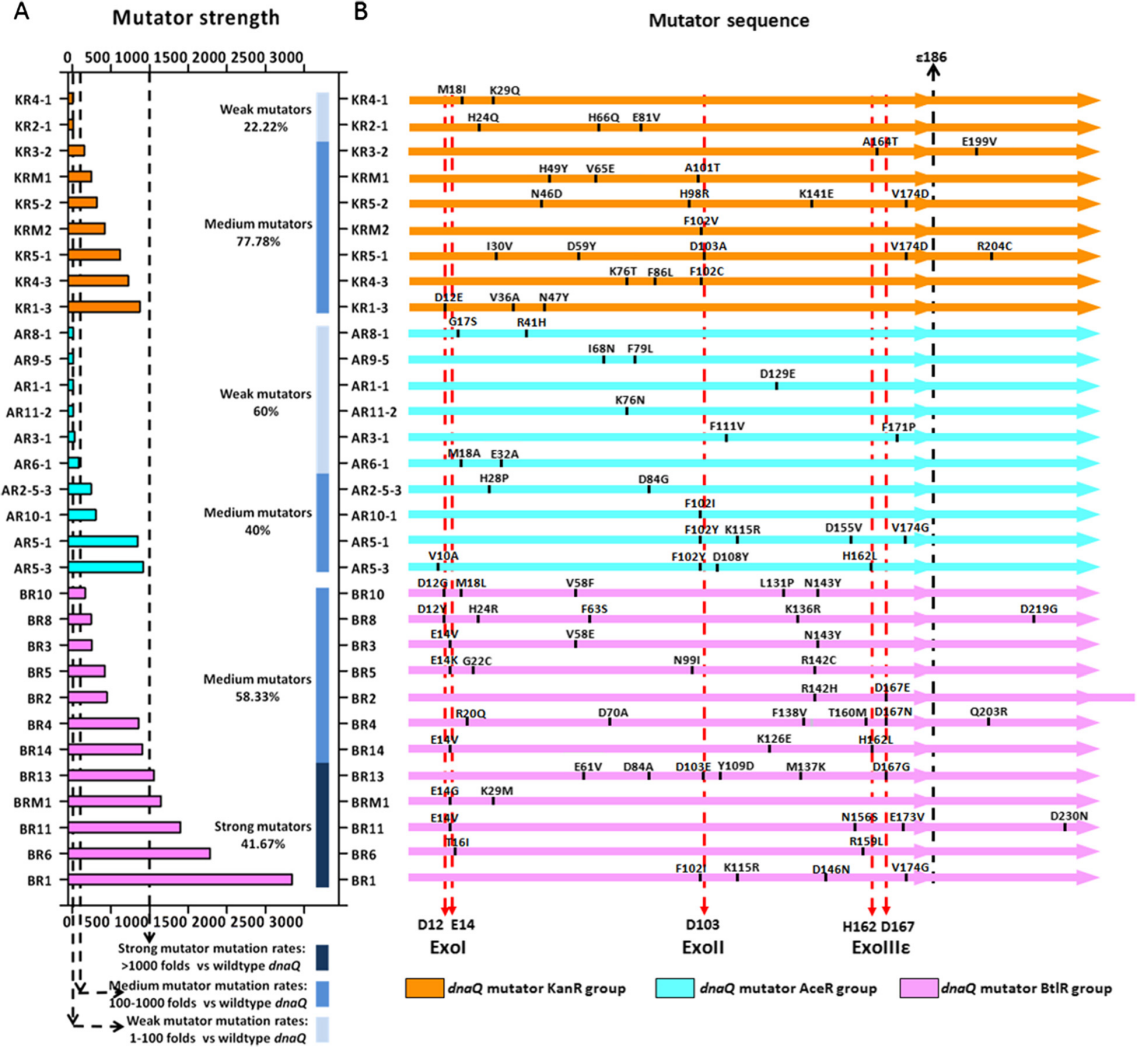
**Strengths and sequences analysis of the *dnaQ *mutators selected from kanamycin stress (KanR group), n-butanol stress (BtlR group), and acetate stress (AceR group). (A)** Mutator strengths of the *dnaQ* mutators relative to the wildtype gene, based on the frequency calculation of rifamycin resistant colony generation. For better understanding and comparisons, weak mutators, medium mutators, and strong mutators are distinguished with light blue, marine blue, and dark blue. **(B)** Summary of the amino acids substitutions on sequences of all the *dnaQ* mutator genes isolated. The full length of the ϵ subunit sequence is divided into two parts by the black line, pointing to the ϵ186. Three red line marked the three conserved and essential regions for the 3′->5′ exonuclease activities. Amino acids substitutions are labeled with black bars and characters.

Three different *dnaQ* mutants (KR5-2, BR1, and AR6-1), which endowed the host with different mutation rates, were enriched during the evolution process against increased concentrations of kanamycin, n-butanol, and acetate. To investigate whether these enriched *dnaQ* mutants were the optimal that could always be enriched in independent evolution processes, we performed parallel experiments for acquiring kanamycin resistance, n-butanol tolerance, and acetate tolerance. The finally enriched *dnaQ* mutants from each process were isolated, sequenced, and analyzed. In the parallel experiments for acquiring kanamycin resistance, nine different *dnaQ* mutants were isolated from 10 independent evolution processes. This suggests that the evolution process for acquiring kanamycin resistance is not dependent on one preferable *dnaQ* mutant. Similar results were also observed in parallel processes for acquiring n-butanol tolerance (12 parallel experiments) or acetate tolerance (10 parallel experiments). All *dnaQ* mutants isolated were different. Sequences of the *dnaQ* mutants isolated were shown in Figure 5B. As expected, all of the selected mutants endowed the host cells with elevated mutation rates, ranging from 7.7 folds (*dnaQ* KR4-1) to 2839 folds (*dnaQ* BR1) (Figure 5A), which indicated the genetically modified *dnaQ* mutants enriched in GREACE could serve as endogenous mutators to accelerate evolution.

We determined the mutation rates of all 31 *dnaQ* mutants isolated from parallel experiments, and grouped them into three types based on the increment of the mutation rates over that of the wildtype *dnaQ* gene (termed as mutator strength for short): weak mutators (1–100 folds), medium mutators (100–1000 folds), and strong mutators (>1000 folds). As shown in Figure 5A, n-butanol stress tended to select strong mutators. A large proportion (5/12) elevated mutation rates of host cells by over 1000 folds, while no weak mutators were found. Acetate stress tended to select weak mutators, as most (6/10) of them falling into the weak group. Kanamycin stress tends to select *dnaQ* mutants with medium mutation rates, as the majority (7/9) showed medium strengths. This suggested that acquiring desired physiological traits required assistance of mutators with specific mutator strength. Calculation of average strengths of the mutators obtained in acquiring n-butanol tolerance (BtlR group, 959.8-fold increase), kanamycin resistance (KanR group, 375.3-fold increase), and acetate tolerance (AceR group, 249.4-fold increase) also supported this indication. Sequencing analysis revealed a positive relationship between the average mutator strengths and average amino acids substitution numbers in each *dnaQ* mutant. Among the BtlR group, an average of 3.9 amino acids substitutions were found in each mutant, while the substitutions for KanR and AceR group were 2.9 and 2.1, respectively.

Analysis of the locations of the amino acids substitutions promoted understanding of the decreased proofreading activities of the selected *dnaQ* mutants. Many efforts have been made to disclose the relationship between sequence and structure characteristics of the ϵ subunits encoded by *dnaQ* gene [16,36]. The 3′ -> 5′ exonuclease activities of the ϵ subunit was mainly determined by the N-terminal 186 amino acids (designated as ϵ186) [37]. In this study, most of the mutation (88 out of 94) from all the 31 *dnaQ* mutants existed within the ϵ186 region (Figure 5B), demonstrating that ϵ186 played key roles in proofreading. Three conserved regions essential for proofreading, ExoI, ExoII, and ExoIIIϵ [38,39], have been recognized in ϵ186. As expected, a large portion of amino acid substitutions of the selected *dnaQ* mutants were located in or quite close to these three regions, meaning high possibilities to disturb the natural proofreading function of the respective gene products.

## Discussion

The accurate genetic information transfer guarantees the genetic and phenotypic stability of organism, and it requires complex and precise mechanisms on multiple stages [18]. However, these hierarchal and precise mechanisms turn to be serious barriers for engineering complex phenotypes. To overcome such barriers, we developed GREACE, which implements a novel principle “Mutagenesis coupled-with Selection”, and thus enables microbial evolution under stressful conditions in a continuous and efficient way.

As for microbes, expanding offspring genome diversities by elevating replication mutation rates to achieve rapid adaptation and competitive advantages in harsh environments has been discovered and verified both *in vivo*[40-42] and *in silico*[43,44]. Various natural mutator genes have been isolated and analyzed. However, some of them failed to work under some harsh conditions, e.g. high temperatures [45,46]. The GREACE method implants a pool of genetically modified proofreading elements into host cells to act as “evolution accelerator”, thus provides multiple mutators with diverse characteristics which might guarantee accelerated evolution of host cells under diverse conditions.

Accelerating microbial evolution by mutators with elevated mutation rates during genome replication have been reported previously [47-49]. All these studies used a single specific mutator with a certain mutation rate. However, out work provided the first evidence that adaptation to specific conditions prefers accumulation of specific mutators, especially with specific mutator strengths. This finding was partially supported by Loh et.al [50], who found a narrow range of mutation rates (10–47 folds increase) dominated a designed laboratory survival competition. It has also been predicted by mathematical models that microbial survival and adaptation to different selective pressures might require different amounts and types of adaptive mutations, thus different mutagenesis strengths might be preferred [44]. These results benefit understanding and application of evolutionary engineering strategies for strain improvement, and further support the necessity for using GREACE method, which generated a library of mutators. Characterization of the *dnaQ* mutants selected from GREACE under different conditions provided interesting clues to understand the intrinsic mechanisms of this novel approach. A universal *dnaQ* mutant that can effectively improve all physiological traits might not exist, while a group of *dnaQ* mutants may be helpful, as the most preferable *dnaQ* mutants will be enriched during the evolution process.

In comparison with other phenotype-improving approaches generating global disturbances by introduction and maintaining of exogenous plasmids [2,11,12], GREACE endows the microbes with stable and heritable phenotypes through scarless manipulations. Genotype stability and phenotype hereditability of the mutants can be maintained at any stage of evolution when the genetically modified proofreading elements are removed from the evolved mutants, and that provides great convenience for further genetic manipulation and application of mutants, e.g. introduction of the metabolic pathway for products synthesis.

Evolutionary engineering has been widely applied to optimize biofuels-producing related characteristics, especially for improvement of substrates utilization and inhibitors tolerant capacities [51-53]. GREACE provides a powerful new tool for evolutionary engineering, as the continuous and exhaustless genetic diversities generated by GREACE can provide nearly every possible solution while the synchronous selection will direct the most advantageous ones. Besides the cellular tolerance phenotypes engineered in this work, GREACE can also be expected to be applied for improving the metabolic capacities of biofuels in combination with the newly arising evolutionary metabolic engineering approach, which establishes the linkage between products synthesis and cell growth [54-56]. In addition, diverse improved phenotypes in mutants evolved by GREACE could be efficiently integrated in a single strain with multiple improved traits by methods like genome shuffling [1,57]. Hence, GREACE can be considered as a promising strain improving approach with wide application potentials.

## Conclusions

A novel method termed as “Genome Replication Engineering Assisted Continuous Evolution” (GREACE), using “Mutagenesis coupled-with Selection” as core principle was developed to improve microbial tolerance for biofuels production. The GREACE method introduced an *in vivo* mutagenesis mechanism into microbial cells by introducing a group of genetically modified proofreading elements of the DNA polymerase (ϵ subunit encoded by *dnaQ* gene) to accelerate the evolution process under stressful conditions. The genotype stability and phenotype heritability can be stably maintained once the genetically modified proofreading element is removed, thus scarless mutants with desired phenotypes can be obtained.

GREACE was successfully applied to engineer n-butanol and acetate tolerances, two important physiological characteristics for biofuels production, demonstrating potentials of the GREACE method for strain improvement in this area. Furthermore, we discovered that adaptation of microbes to specific stresses prefers specific mutagenesis strengths, which may provide new insights on understanding and application of evolutionary engineering for strain improvement.

## Methods

### Strains and culture conditions

*E. coli* DH5α (TAKARA) was used for plasmids construction, phenotype evolution, and mutation rates evaluation. *E. coli* cells were grown aerobically in Luria-Bertani medium at 37°C, unless there are special instructions. Antibiotics and kanamycin, n-butanol, and acetate were supplemented as required.

### Plasmid and library construction procedures

The native *dnaQ* gene and promoter fragment were both amplified from the *E. coli* DH5α genomic DNA with Phusion DNA polymerase, using primer pairs dnaQ-F/dnaQ-R, and dnaQProm-F/dnaQProm-R, respectively (Additional file 1: Table S1). The *dnaQ* fragment was cloned into the EcoRI and HindIII sites in pUC18 and the *dnaQ* promoter sequence was used to replace the *lac* promoter between the BspQI and EcoRI sites. The final plasmid was named as pQ-dnaQ.

Error-prone PCR was employed to construct a *dnaQ* mutant library, with primer pairs of dnaQ-EP-F/dnaQ-EP-R (Additional file 1: Table S1). A standard error-prone PCR protocol was taken, and the mutation rate was controlled by adjusting concentrations of manganese and magnesium ions. The products was purified, digested and inserted into EcoRI and NdeI sites of the pQ-dnaQ plasmid to replace the wild type *dnaQ* gene. The ligation system was transformed into *E. coli* DH5α cells. After cultivation on agar plates, about 10^6^ transformants were obtained and scrapped off, and then the plasmids were extracted to generate a library named as pQ-lib. All enzymes used for DNA manipulation are from NEB.

### Evolution and phenotype selection

*E. coli* DH5α cells that were respectively transformed with pQ-lib, pQ-dnaQ, and pUC18 were transferred into fresh LB medium containing 100 μg/ml ampicillin (Amp100 for short) and grown overnight. Then the broth was serially transferred under gradually increased stress conditions. All of the evolution experiments in this part were performed with 10 ml LB media supplemented with Amp100 and serial concentrations of kanamycin, n-butanol or acetate, and cultivated at 37°C, unless there are special instructions. To evaluate cell densities, OD_600_ was monitored by microplate reader at 600 nm with a sample volume of 200 μl.

(i) Kanamycin resistance: 300 μl of overnight culture broth of the transformants was inoculated into 10 ml LB medium containing Amp100 and Kan15, and cultivated for 2 days. Then 300 μl of the culture broth (with an OD_600_ value adjusted to 0.2 by fresh LB medium) was transferred to LB medium containing Amp100 and Kan100, and cultivated for 2 days. The transfer and selection processes were repeated to LB media containing Kan200 and Kan300.

On each selection level, about 10^8^ cells from the broth were spread on LB agar plates containing Amp100 and kanamycin concentrations of the next selection level, and cultivated for colony counting. For example, cells from Kan100 broth would be spread on plates containing Kan200.

On each selection level, the culture broth was spread on LB agar plates containing Amp100 and incubated overnight. 30 single colonies were selected randomly and the *dnaQ* mutants carried were isolated and sequenced.

(ii) n-Butanol tolerance: evolution process for n-butanol tolerance of *E. coli* was similar to that described for kanamycin resistance. After a pre-cultivation in 0.5% of n-butanol, culture broth was stepwise transferred to LB media containing Amp100 and n-butanol with gradually increased concentrations from 0.625% to 1.25%. Inoculation volume and cell densities for the three groups were adjusted to the same. On each n-butanol concentration, three transfers were performed before inoculation into a higher level. Each cultivation was performed for 48 hours. Tubes used in n-butanol tolerance evolution were sealed off with parafilm to avoid n-butanol evaporation.

(iii) Acetate tolerance: evolution process for acetate tolerance of *E. coli* was similar that described for n-butanol tolerance. Cultivations were performed in serial acetate concentrations of 0.06%, 0.075%, 0.09%, and 0.105%.

### Plasmid elimination

The plasmid in the finally evolved strain was eliminated by serial transfer in LB medium without ampicillin but supplemented with the corresponding selective pressure (e.g., high concentrations of kanamycin or butanol or acetate). An appropriate amount of culture was spread on the agar plate with the same concentration of selective pressure before each transfer for colony visualization. Thirty single colonies were streaked on agar plate with ampicillin and those with ampicillin sensitivity were regard as the plasmid-cured ones. Typically 5–10 transfers are sufficient to isolate plasmid-cured strain maintaining the evolved phenotypes.

### Growth assay

To evaluate tolerance of the GREACE generated cells, growth of the finally isolated mutant strains were analyzed under serial concentrations of n-butanol or acetate and compared with that of the wildtype *E. coli* strain. For growth assay, *E. coli* cells were cultivated overnight at 37°C in LB medium, and then diluted at a ratio of 1:100 into fresh LB media added with serial concentrations of n-butanol or acetate. Cultivations at 37°C, 200 rpm were performed for 48 hours before calculation of OD_600_ to evaluate the cell densities.

### Shock experiment

To explore tolerance and stability of *E. coli* BT12 strain under extreme lethal n-butanol stress, shock experiments were performed with 2% (vol/vol) of n-butanol. Procedure of the shock experiment was similar with previously introduced [58]. *E. coli* BT12 and *E. coli* DH5α cells were cultivated overnight at 37°C in LB medium, and then diluted at a ratio of 1:100 with fresh LB medium and grown at 37°C to OD600 of 0.3~0.5. The cultures were then diluted with LB to OD_600_ of 0.3, and n-butanol was added to final concentrations of 2% (vol/vol). After incubation at 37°C for 1 hour, the cultures were serially diluted, plated on LB agar plates and cultivated at 37°C overnight for photographing.

### Determination of mutation rate

All of the selected *dnaQ* mutators were retransformed into *E. coli* DH5α strain with clean genetic background to determine the mutation rate. We calculated generation frequency of rifamycin resistant mutant cells to evaluate the mutation rates of the host cells, a method that has been applied widely in types of microbes [59,60]. *E. coli* cells transformed with specific *dnaQ* mutant were spread on LB agar plates and cultivated overnight in 37°C. Three colonies were inoculated into fresh LB medium containing Amp100 and grown overnight. Approximately 10^7^ cells from the cultivation broth were spread on LB agar containing Amp100 with or without 100 μg/ml of rifamycin, and incubated in dark for 2 days. The rifamycin resistant colonies and total colonies were counted, and CFU data were used for determination of mutation rates.

## Abbreviations

GREACE: Genome replication engineering assisted continuous evolution; PE: Proofreading element; KanX: X μg/ml kanamycin; Amp100: 100 μg/ml ampicillin; LB: Luria-Bertani.

## Competing interests

The authors declare that they have no competing interests.

## Authors’ contributions

GDL and ZC designed and performed the research. GDL, ZC, and YL analyzed the data. GDL, ZC, YL, and YHM wrote the manuscript. YL and YHM supervised the project. All authors read and approved the final manuscript.

## Supplementary Material

Additional file 1 Table S1 Primers used for plasmids and library construction in this work. **Figure S1.** Growth assay of *E. coli* strains carrying the pQ-dnaQ-KR5-2, pQ-dnaQ, and pUC18 plasmids in kanamycin concentrations of 15 μg/ml (A) and 100 μg/ml (B). **Figure S2.** Growth assay of the *E. coli* KR1 (pQ-dnaQ-KR5-2) and *E. coli* KR1 under kanamycin stress with (A) or without (B) Amp100. **Figure S3.** Shock experiment by 2% n-butanol of the n-butanol tolerant strain *E. coli* BT12. **Figure S4.** Growth assay of the n-butanol tolerant strain *E. coli* BT12 in serial n-butanol concentrations with or with pre-treatment. Click here for file
