# Supplementary material for: Genome replication engineering assisted continuous evolution (GREACE) to improve microbial tolerance for biofuels production
Source: Biotechnol Biofuels. 2013 Sep 27;6:137. doi: 10.1186/1754-6834-6-137 (PMC3856464; doi:10.1186/1754-6834-6-137)
Supplement: Additional file 1 Table S1 — Primers used for plasmids and library construction in this work. Figure S1. Growth assay of E. coli strains carrying the pQ-dnaQ-KR5-2, pQ-dnaQ, and pUC18 plasmids in kanamycin concentrations of 15 μg/ml (A) and 100 μg/ml (B). Figure S2. Growth assay of the E. coli KR1 (pQ-dnaQ-KR5-2) and E. coli KR1 under kanamycin stress with (A) or without (B) Amp100. Figure S3. Shock experiment by 2% n-butanol of the n-butanol tolerant strain E. coli BT12. Figure S4. Growth assay of the n-butanol tolerant strain E. coli BT12 in serial n-butanol concentrations with or with pre-treatment. [file 1754-6834-6-137-S1.docx]

**Additional file 1**

Table S1. Primers used for plasmids and library construction in this work

| Primers | Sequences | Use |
| --- | --- | --- |
| dnaQ-F | GGGCCGGAATTCTATGAGCACTGCAATTACACG | Clone of *dnaQ* gene from *E. coli* DH5α |
| dnaQ-R | GGGCCGAAGCTTTGCTGCAAAAATCGCCCAAGT |  |
| dnaQProm-F | TTAATGCTCTTCCGCTTAGCGCGACAATAGCGGCCATC | Clone of the native promoter for *E. coli* *dnaQ* gene |
| dnaQProm-R | GGACCTGAATTCGTCATAGCGGTCATTTATGTCAGA |  |
| dnaQ-EP-F | CGTATGTTGTGTGGAATTGTG | Error prone PCR to generate the *dnaQ* mutants library pQ-lib |
| dnaQ-EP-R | GGAATTCATATGCCAGCTGGCGAAAGGGGGATG |  |


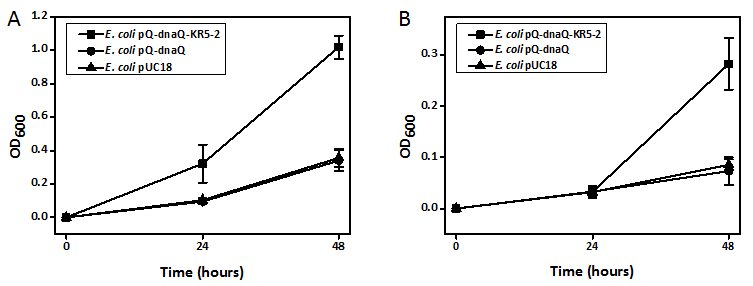


Figure S1. Growth assay of *E. coli* strains carrying the pQ-dnaQ-KR5-2, pQ-dnaQ, and pUC18 plasmids in kanamycin concentrations of 15 μg/ml (A) and 100 μg/ml (B). For all of the three strains, 3 colonies were picked and incubated to fresh LB medium containing Amp100, and cultivated at 37^o^C overnight. 100 μl broths were incubated to 10 ml LB medium containing Amp100 and Kan15 (A) or Kan100 (B), and cultivated for 48 hours. Cell densities were calculated by OD_600_.


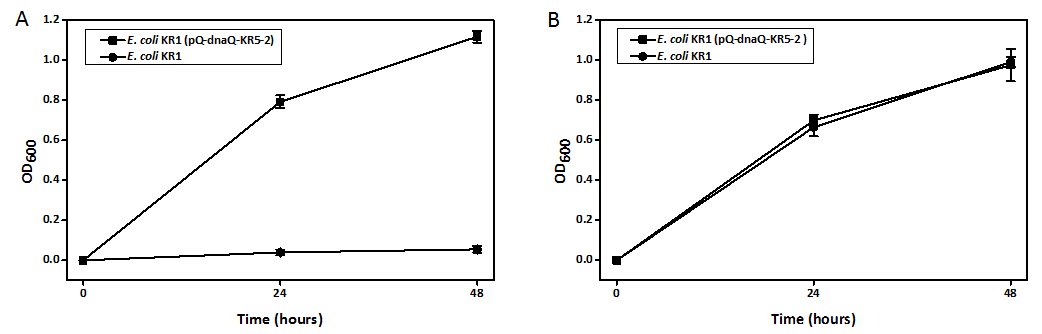


Figure S2. Growth assay of the *E. coli* KR1 (pQ-dnaQ-KR5-2) and *E. coli* KR1 under kanamycin stress with (A) or without (B) Amp100. 100 μl overnight culture of *E. coli* KR1 (pQ-dnaQ-KR5-2) and *E. coli* KR1 were respectively incubated into LB media containing Kan300 with or without Amp100, and cultivated at 37 ^o^C for 2 days. Cell densities were calculated by OD_600_.


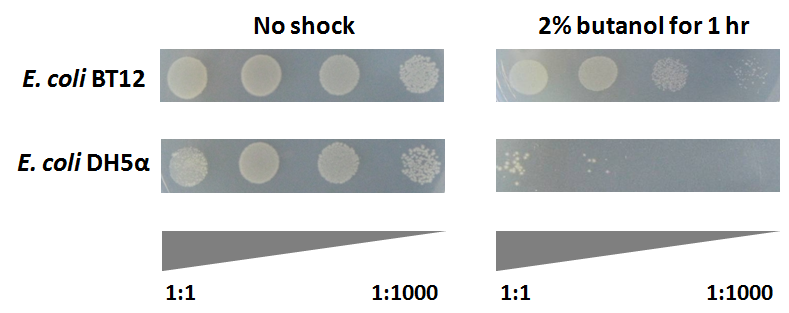


Figure S3. Shock experiment by 2% butanol of the butanol tolerant strain *E. coli* BT12. Stabilities and viabilities of *E. coli* BT12 and *E. coli* DH5α under extreme lethal butanol concentrations (right) and no butanol stress (left) were tested and compared. Triangles below each panel indicate serial dilutions of plated cells (1:1 to 1:1000, from left to right)


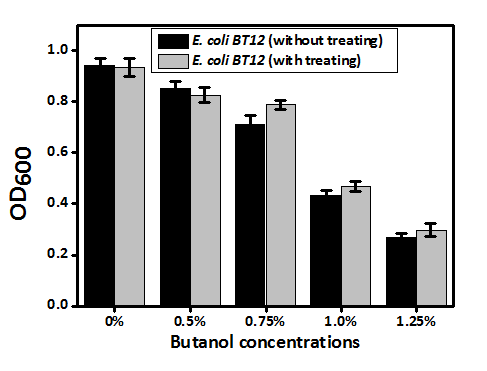


Figure S4. Growth assay of the butanol tolerant strain *E. coli* BT12 in serial butanol concentrations with or with pre-treatment. *E. coli* BT12 previously stored at -80 ^o^C for 2 weeks was activated and cultivated in LB medium without butanol stress for over 30 passages and labeled as *E. coli BT12* (with treating), while the *E. coli BT12* stored under butanol stressful conditions was termed as *E. coli BT12* (without treating).
